# Interorganizational Mechanisms for Developing and Implementing Clinical Decision Support Systems in Primary Care: Exploratory, Qualitative Case Study

**DOI:** 10.2196/83084

**Published:** 2026-03-05

**Authors:** Jesse J M M Santema, Jeroen D H van Wijngaarden, Eric G Hiddink, Fleur Deken, Maaike Kleinsmann, Hendrikus J A van Os

**Affiliations:** 1 Industrial Design Engineering, Delft University of Technology, Mekelweg 5, Delft, South Holland, 2628 CD, The Netherlands, 31 612815254; 2 Health Services Management & Organization, Erasmus School of Health Policy and Management, Rotterdam, South Holland, The Netherlands; 3 National eHealth Living Lab, Department of Public Health & Primary Care, Leiden University Medical Center, Leiden, South Holland, The Netherlands; 4 Health Base Foundation, Houten, Utrecht, The Netherlands; 5 School of Business and Economics, Knowledge, Information and Innovation, Vrije Universiteit Amsterdam, Amsterdam, North Holland, The Netherlands; 6 Department of Cardiology, Leiden University Medical Center, Leiden, South Holland, The Netherlands; 7 Department of Psychiatry, University Medical Center Utrecht, Utrecht, Utrecht, The Netherlands

**Keywords:** clinical decision support systems, interorganizational collaboration, mechanisms of change, multilevel stakeholder involvement, integration of people, integration of technology, integration of business, iterative development and implementation, process-level analysis

## Abstract

**Background:**

Clinical decision support systems (CDSS) have the potential to improve patient safety and reduce costs in primary care. However, CDSS adoption remains limited due to development and implementation challenges. CDSSs are complex interventions involving multiple interacting components that require technological innovation and behavioral and organizational change. Additionally, the primary care context is considered a complex system with high care demand, fragmented structures, and many independent yet interdependent organizations. Established determinant frameworks for implementing and scaling up complex health care interventions support the identification of implementation determinants. However, they offer limited guidance on the underlying processes of these determinants, such as the implementation processes involved in complex interorganizational collaboration in primary care.

**Objective:**

This study examined how an interorganizational collaboration in Dutch primary care (*Gezonde zorg, Gezonde regio* [*GzGr*]) achieved an iterative CDSS development and implementation. We aimed to identify the mechanisms that supported the collaboration in overcoming challenges.

**Methods:**

We performed an exploratory process-level case study. Data were collected through 15 semistructured interviews. The nonadoption, abandonment, scale-up, spread, and sustainability framework was used to ensure comprehensive topic coverage during the interviews, but not as an analytical framework. We triangulated the interviews with internal and external documents and expert input. Using a thematic, inductive approach, we developed a chronological overview of the collaboration and identified mechanisms offering insights into how GzGr navigated complexity in the development and implementation of CDSS.

**Results:**

We identified two mechanisms: (1) enacting an interorganizational value model and (2) iterative, co-creative experimentation. First, GzGr was driven by a coalition of the willing (ie, individuals willing to take an extra step), with shared goals that prioritized collective benefit while respecting organizational values. They established shared principles that translated the broad GzGr mission into concrete CDSS development choices, while also guiding strategic expansion by involving mission-aligned, innovative organizations. Second, after initial prototypes, GzGr established an iterative learning and improvement experimentation for both the technology and the collaboration. This process allowed for rapid feedback, validation of added value, and ongoing refinement. Additionally, this experimentation approached the development and implementation phase as a continuous process involving multistakeholders, supporting both the technology and the collaboration.

**Conclusions:**

This study identified 2 mechanisms that sustained interorganizational collaboration and CDSS development. These mechanisms connected collaborative and technical changes across people, technology, and organizational levels, enabling technological viability, stakeholder value, and multilevel support. The mechanisms operated both within and between organizations through iterative cycles of development and implementation. Practical implications include involving multilevel, innovative, and influential stakeholders; maintaining alignment through an orchestrating actor; and adopting an iterative approach between development and implementation. Our findings extend existing determinant frameworks by offering process-level insights into how such mechanisms help overcome challenges in the development and implementation of CDSS within interorganizational collaborations.

## Introduction

Clinical decision support systems (CDSS) hold the potential to improve patient safety, diagnostic accuracy, and clinical decision-making and reduce health care costs [1]. CDSSs achieve these aims by using rule-based or artificial intelligence (AI) algorithms to deliver patient-specific alerts, reminders, and dosage support, especially in primary care [2-4]. Despite its potential, CDSS adoption remains limited due to interdependent development and implementation challenges [1,5-7], particularly in primary care [2,8,9]. These challenges partially stem from the complexity of CDSS, as they involve multiple interacting components, often necessitate behavioral changes from end users, and affect clinical and economic outcomes [10,11]. The complexity of CDSS also results in interconnections between users and their social and organizational context.

Traditional implementation approaches assess interventions under controlled conditions before applying them in practice [10,12-15]. This approach can lead to unintended consequences during real-world implementation due to the intervention’s complexity [14]. Therefore, digital health and innovation scholars advocate for real-world implementation with iterative cycles consisting of development and implementation that proceed [10,12-14,16-18]. These iterative cycles allow contextual adaptation and gradual refinement, which improves implementation outcomes and mitigates unintended consequences [13,14]. Examples of models for an iterative process are the Centre for eHealth and Wellbeing Research roadmap, UK Medical Research Council guidelines, and the nonadoption, abandonment, scale-up, spread, and sustainability (NASSS) framework [10,12,13,18]. These models provide valuable insights into determinants for the implementation of digital health interventions in single organizations (while recognizing the wider contextual factors influencing implementation). However, these models pay less attention to the interactions among determinants and processes underlying these determinants [14,19].

**Figure 1.**
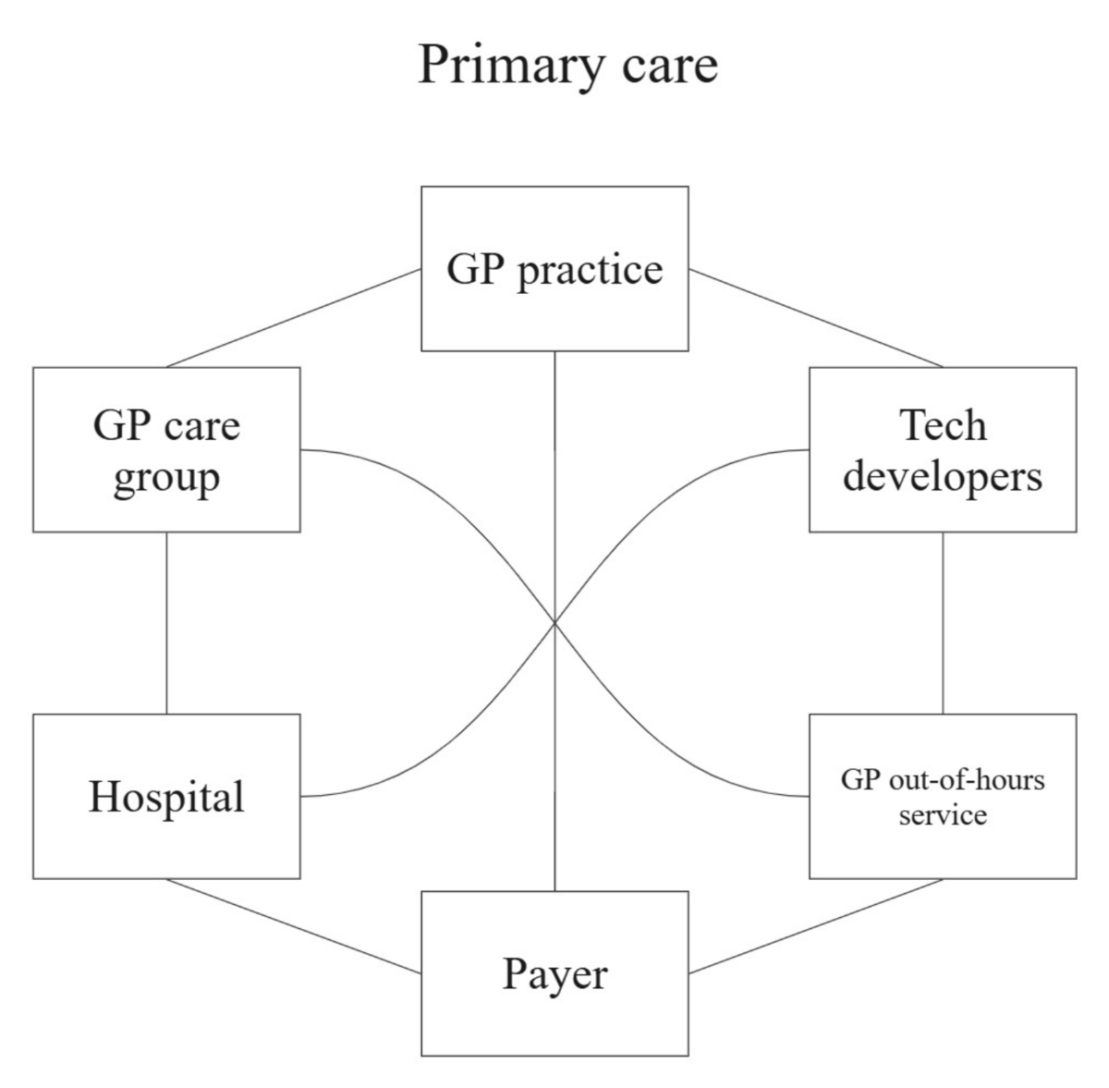
Simplified overview of 6 independent organizations in Dutch primary care and their connections. The connecting lines highlight complexity. Every organization must interact with every other organization for the continuity and accessibility of care. GP: general practitioner.

Insights into these underlying processes are especially relevant in the context of CDSS in primary care, where both the intervention and the setting are inherently complex. Primary care in the Netherlands involves many independent yet interdependent organizations, such as general practices, care groups, payers, and technology developers [2,14,20,21]. These stakeholders differ in roles, interests, and values owing to separate financing structures, although they must collaborate across organizational boundaries to ensure continuity and accessibility of care. This results in a highly fragmented and complex care landscape. Stakeholders and their interactions are illustrated in Figure 1. Research suggests that a context with multiple independent and interdependent organizations calls for interorganizational collaboration to enable iterative development and implementation [11,16]. Such collaboration aims for coordinated efforts between organizations working toward a shared goal [22,23]. However, these studies do not elaborate on how an interorganizational collaboration can be established in practice.

We built on prior determinant research conducted in single-organization implementation contexts [10,12,13,18], which emphasized the need for an iterative CDSS development and implementation process. Our study extended this line of research by examining how challenges encountered during development and implementation were addressed in an interorganizational primary care collaboration. Specifically, we focused on identifying mechanisms that explain the underlying processes through which interventions are developed and implemented [24]. This focus is particularly relevant in complex settings such as primary care. Our research question was as follows: What mechanisms facilitate overcoming challenges in the development and implementation of CDSS within an interorganizational collaboration in primary care? We aimed to identify mechanisms that enabled CDSS development and implementation within an interorganizational collaboration in primary care. We conducted a qualitative process analysis of a Dutch primary care collaboration, allowing us to focus on how challenges and actions developed over time and formed mechanisms.

## Methods

### Study Design

We performed an exploratory case study to identify which mechanisms enabled *Gezonde zorg, Gezonde regio* (GzGr) to successfully develop and implement CDSS in Dutch primary care [25]. We define mechanisms in the context of this paper as sequential patterns of actions that explain how change occurs, both between and within organizations in relation to technological interventions. These actions represent how GzGr created the conditions and behaviors needed to cope with challenges that occurred over time in the development and implementation of complex digital health solutions in complex settings. The sequential aspect emphasizes how actions build on previous actions or challenges over time, indicating that their order may be causally related to their final effect. To identify how, why, and when mechanisms were applied, we used qualitative process analysis by tracing actions and decisions over time [26-28]. While the mechanisms captured what drove change*,* our accompanying narrative illustrated how these processes unfolded in practice. This study was designed and reported in line with the COREQ (Consolidated Criteria for Reporting Qualitative Research) checklist (Checklist 1) [29].

### Case Study Selection

We researched “Healthy care, Healthy region” (in Dutch: *Gezonde zorg, Gezonde regio* [*GzGr*]). We identified GzGr as a unique case study via field exploration through researchers’ networks and via internet searches. Case selection was based on the following criteria: (1) successful regional implementation of a CDSS across multiple primary care practices, (2) full integration in primary care workflow, including IT integration; and (3) collaboration involving all relevant stakeholders in primary care.

### Case Description

**Figure 2.**
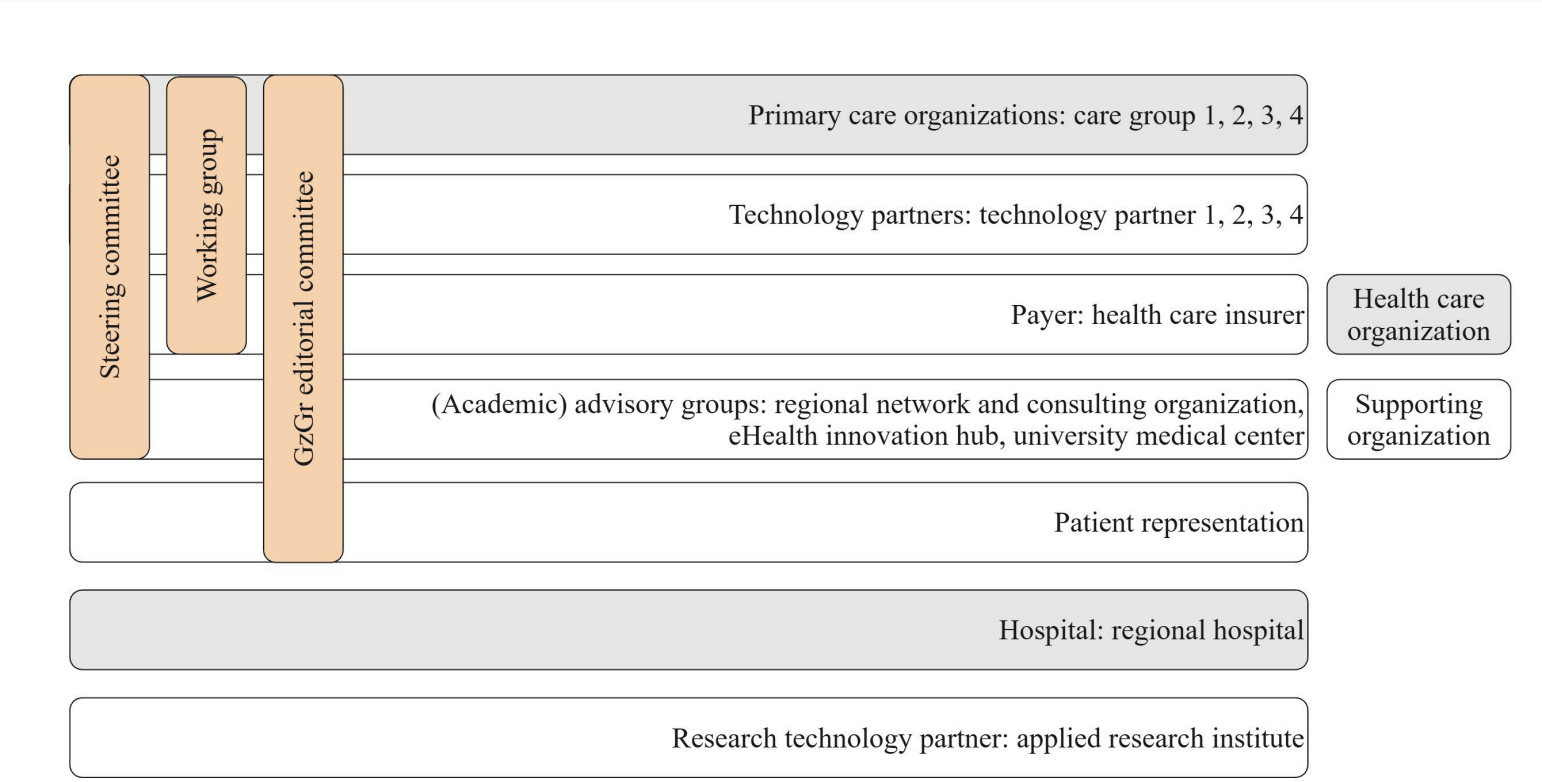
Visual representation of the GzGr structure, with health care and supporting organizations (represented by the horizontal squares). The vertical squares represent the governance structure. GzGr: *Gezonde zorg, Gezonde regio*.

The GzGr initiative involves a diverse range of organizations: general practitioner (GP) practices, regional primary care organizations, a regional hospital, a health care insurance firm, data processing firms, technology and content development firms (collectively referred to as technology partners), and advisory groups responsible for implementation support and technology evaluation [30]. Figure 2 provides a visual representation of the GzGr structure. GzGr developed multiple rule-based and data-driven CDSS to provide real-time alerts to GPs regarding patient health risks [31]. The steering committee, consisting of executives from key partner organizations, sets the strategic direction and oversees key decisions. The working group, consisting of health care purchasers and innovation managers from the care groups, manages day-to-day operations and the collaboration and advises the steering committee. The editorial committee consists of end users, technology developers, and representatives from the health care insurance firm and is responsible for identifying widely experienced problems and overseeing CDSS development.

### Data Collection

We collected multiple sources of data, including interviews and documents, aiming for data triangulation to increase the validity of the data [32]. In total, we recruited and interviewed 15 unique respondents from December 2021 to September 2022 by JDHvW. Interviews were conducted until data saturation was reached, meaning that interviews confirmed findings rather than yielded new findings [33]. Data saturation determined our study size. We interviewed at least one participant from each partner organization involved in GzGr. An overview of participants is given in Multimedia Appendix 1. Our eligibility criteria for participants were their (1) active involvement in the operational development of the CDSS, by working at one of the technology partners, at a general practice or a care group (defined as an organization that coordinates and supports GPs with administrative burden); and/or (2) involvement in the strategic decision-making of GzGr, either through participation in the steering committee or by holding an executive mandate within an organization.

We sampled the respondents using snowball sampling and purposive sampling and used the networks of participants, partner organizations of GzGr, and researchers [34]. JDHvW contacted participants by email through a GzGr contact person, after which additional participants were identified by previously interviewed respondents. Although this approach introduced potential selection bias at first, snowball sampling helped recruit a diverse and representative group. No participants declined participation.

We followed a semistructured interview approach to examine which mechanisms were used by GzGr to ensure progress in development and implementation. We composed an interview topic guide based on the NASSS framework [12]. Topics were based on the following domains: intervention features, the expected value, adopter readiness, the broader context, adaptability over time, and the interplay among domains. We omitted the “condition” domain, as the developed CDSS were designed to apply across multiple clinical conditions. Furthermore, we asked about involved stakeholders, organizational characteristics, and the development process. We explored how GzGr addressed related challenges in these domains to identify underlying mechanisms, which formed the basis for identifying mechanisms.

Interviews were conducted via video call. Each interview lasted between 45 and 60 minutes, and the respondent(s) and JDHvW were present. The interviews were audio recorded and afterward transcribed verbatim. Transcripts were made available to respondents for review upon request. Moreover, we collected internal and external documents of the organizations via the participants and the internet, from September 2023 to January 2024. All documents were stored in Atlas.ti (ATLAS.ti Scientific Software Development GmbH) for analysis.

### Data Analysis

To identify mechanisms that facilitated overcoming challenges in the development and implementation of CDSS, our data analysis consisted of multiple steps, namely (1) composing a chronological overview; (2) thematic and inductive coding to identify mechanisms; (3) performing a member check; and (4) constructing a conceptualized narrative. We used Atlas.ti 9 to support the coding process. Further elaboration on the coding process and the codebook is given in Multimedia Appendix 2.

1. We created a chronological overview of challenges and actions related to development, implementation, or collaboration encountered or undertaken by GzGr to provide deeper insight into how the stakeholders developed and implemented CDSS. The chronological overview is given in Multimedia Appendix 3.
2. Next, we conducted an in-depth analysis to identify mechanisms, consisting of patterns of actions taken by GzGr. We used thematic analysis and inductive coding to reveal new insights and underlying patterns in the data [35,36]. We performed a second round of open coding of the transcripts. During open coding, codes that had been generated in the earlier transcripts were applicable to later interviews. At that stage, no new codes were introduced, which indicated that we reached code saturation. These codes were used for composing a codebook (see Multimedia Appendix 2) and for coding all the transcripts. Then, we identified 2 mechanisms, by linking challenges and determining the sequential patterns of actions that, according to participants, had led to the successful development and implementation of CDSS. Mechanism 1, enacting an interorganizational value model, is defined as aligning independent yet interdependent organizational goals and values into regional goals and values and translating them into practical actions. The mechanism 2, iterative, co-creative experimentation, is defined as continuously, jointly developing and adapting solutions with stakeholders, treating development and implementation as one interconnected, ongoing process. In Multimedia Appendix 3, we further elaborated on the link between challenges and actions and how the mechanisms emerged or evolved over time.
3. We performed member checks with the steering committee of GzGr to reach consensus on the mechanisms deployed. Additionally, EGH and HJAvO, who participated in the GzGr collaboration as (scientific) experts, supported the interpretation of qualitative data.
4. The final step in the data analysis was constructing a narrative of the challenges encountered and the actions taken by GzGr, supported by quotations of the participants. At the end of every period, we conceptualized the sequential patterns of actions that formed a mechanism. Furthermore, we bundled the sequential patterns of actions per mechanism in Table 1. In the discussion, we compared these findings with prior work.

**Table 1.** Mechanisms consisting of sequential patterns of actions to develop and implement complex digital health solutions in complex settings.

Mechanism	Sequential processes
Enacting an interorganizational value model	Forming a coalition of the willing Defining and aligning on principles for organization selection and development early in the process (ownership, pricing, co-creation, transparency, guidelines, embedding in workflows) Streamline decision-making and maintain focus during development due to aligned principles
Iterative, co-creative experimentation	Co-creation in structured feedback loops between end users, developers, and decision-makers Taking ownership of communication by the collaboration Technological adjustments combined with measurable scientific outcomes

### Ethical Considerations

This study was conducted in accordance with ethical guidelines for qualitative research involving human participants. Ethical approval was obtained from the Research Ethics Review Committee of the Erasmus School of Health Policy & Management (reference number 21‐014), ensuring compliance with relevant regulations on data protection and participant rights.

Additionally, participants signed an informed consent form before participating in interviews, which stated the goal of the research, the researchers, and the interview; privacy measures (including pseudonymizing data); and confidentiality measures (raw data available upon reasonable request) and included consent for audio recording. No identifying information or images of individual participants are included in this manuscript or its supplementary materials. Participants did not receive any compensation.

## Results

### Overview

In this section, we described the chronological sequence of the development and implementation challenges and actions. Table 2 summarizes the main challenges faced by GzGr and the resulting actions that led to the development and implementation of CDSS. In retrospect, we distinguished 3 periods, each with distinct challenges and focus. The chronological overview in Multimedia Appendix 3 is a more elaborated version of these challenges, actions, and their connection to the mechanism and provides a brief overview of the timeline.

**Table 2.** Summary overview of challenges and actions across 3 periods that contributed to enacting an interorganizational value model and to applying iterative, co-creative experimentation.

Period, challenge, and actions	Mechanism leveraged
2010‐2014: forming a coalition of the willing	
Staff shortages within GzGr^a^	
Started collaboration with influential individuals	1
Exploring technological needs, unexpected outcomes, and collaboration stopped	
Research institute measured the baseline population funding	1
Engaged, innovative alignment is difficult	
Composing coalition of the willing and aligning principles for collaboration expansion	1
Engaged, innovative alignment is difficult	
Technology partner 1 and regional network and consulting organization joined the collaboration	1
Mission changed: enhancing preventive care by developing CDSS^b^	1
Technology partners 2 and 3 joined the organization based on aligned principles	1
First pilot CDSS development	1
Not all organizations were innovative	
Shift in focus to involving innovative GP^c^ practices and organizations	1
2015‐2017: develop understandable CDSS	
Engaged, innovative alignment is difficult	
Establishing CDSS development principles	1
Building understandable CDSS	1
Lack of workflow integration, PDF notifications for CDSS	
Technology partner 4 joined, ensuring integration of CDSS in GP information system	1
Six knowledge rules deployed, five in development	1, 2
2018‐2021: Implement CDSS in daily practice	
Use of CDSS low	
More focus on implementation	2
Communication responsibility of individual care groups	
GzGr improved information material, developed training, and informed community	2
Focus shift to implementation	
Established editorial committee for identifying implementation problems	2
Focus shift to implementation	
Implementation staff assigned, improving the digital skills of health care professionals	2
Improve CDSS	
Testing CDSS prototypes and retrieving feedback from GPs	1, 2
Developed dashboard with insights into the use of CDSS	2
Strategic organizational dialogues about opportunities for individual organizations and GzGr	1, 2

a GzGr: *Gezonde zorg, Gezonde regio* (Healthy care, Healthy region).

b CDSS: clinical decision support system.

c GP: general practitioner.

### Period 1 (2010-2014): Forming a Coalition of the Willing

In this first period, we explain how GzGr started their collaboration with a mission, formed a mission-driven coalition of the willing, and established principles for expanding the collaboration.

The GzGr collaboration was initiated by 3 senior influential individuals in health care. One was a senior executive at the largest regional health care insurer, securing long-term funding. The other two were executives from a primary care group and a regional hospital, providing managerial commitment. These individuals were considered influential in the region because they could allocate resources, influence organizational decisions, and mobilize support (financial and people). The aim was to provide more efficient care to maintain regional health care accessibility, with a focus on prevention, the use of routine data, and in line with shared savings funding models. In their view, delivering future-proof care required close collaboration between care organizations and funders to foster joint responsibility for a specific population. As an initial attempt toward operationalizing this mission, a research-focused technology partner explored the feasibility of population-based funding. However, it became evident that this organization lacked expertise in operationalizing the outcomes, posing a challenge for GzGr, leading GzGr to stop the collaboration.

Realizing the long-term mission to aim for more efficient care and involving partnering organizations was also experienced as challenging for GzGr; therefore, they decided to refocus their efforts on a more short-term, actionable mission: enhancing preventive care interventions in GP practices by developing and implementing CDSS. This system would leverage routine care data to support GPs in patient monitoring and the early detection of health risks.

To realize the development, GzGr invited 2 new individuals from organizations to join the collaboration: an innovation manager working at technology partner 1, contributing to clinical content and CDSS development, and a project manager from the regional network and consulting organization. According to respondents, the addition of these 2 organizations was key in translating GzGr’s broad mission into an actionable, concrete CDSS. Together, these 5 individuals brought extensive professional networks, complementary expertise, and executive backing from their organizations:

*Our success lies in the combination of having people who deeply understand the data and work with it daily, others who know the primary process well, a few executives who are genuinely enthusiastic about this, and a healthcare insurer that recognizes its importance.*

[Professor of data science, technology partner 1]

More importantly, respondents emphasized that these individuals consistently prioritized the shared, collaborative mission. At the same time, they ensured alignment with their own organizational interests, without aiming to maximize individual organizational gain. GzGr referred to the individuals as a “coalition of the willing,” marking the emergence of a mission-driven collaboration within GzGr:

*Based on knowing a few enthusiastic people, being aware of the expertise available, having a shared goal, and the motivation to pursue it: we just went ahead and did it. We developed a proof of concept and demonstrated that it works. Even stakeholders said: This will accelerate things.*

[Policy adviser, national health care insurance umbrella organization]

To make progress, GzGr recognized the need for a data infrastructure to support CDSS implementation across GP practices and enable data integration and evaluation. This meant that additional technology partners were needed. Identifying technology partners that aligned with GzGr’s mission and collaborative approach proved challenging. Therefore, GzGr formed principles for organization selection and task allocation: willingness to experiment, previous experience in CDSS development, knowledge of legal and regulatory standards, possession of appropriate certifications, a willingness to work at a cost-plus model (ie, a pricing approach where providers are reimbursed based on actual costs plus a fixed margin), and agreeing upon the shared mission. These principles ensured that new participating organizations aligned with GzGr’s chosen direction, allowing flexibility in how the mission was executed but preserving its overall purpose, which prevented mission drift and delay in progress.

**Figure 3.**
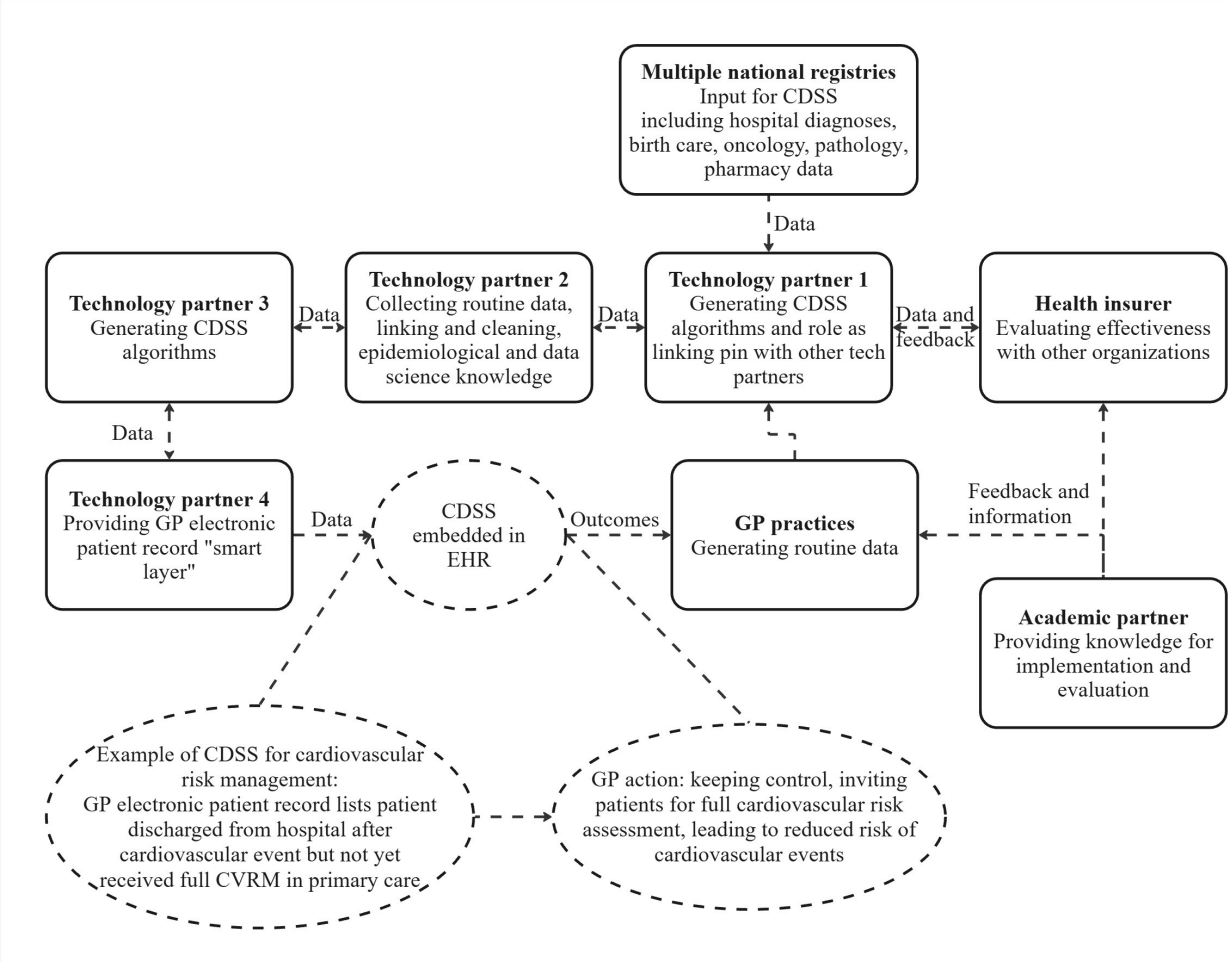
Organizations involved in developing and implementing CDSS within GzGr, showing data integration, embedding in GP records, and continuous evaluation. The squares represent participating organizations, the circles represent an example of CDSS, and the arrows represent the data infrastructure. CDSS: clinical decision support system; CVRM: cardiovascular risk management; EHR: electronic health record; GP: general practitioner.

Through technology partner 1’s network, technology partners 2 and 3 joined. Together, GzGr began developing CDSS that could be implemented at scale. One of the first successful pilots was a data-driven CDSS for cardiovascular pharmacotherapy, identifying cost-effective treatment options with equal or better outcomes. Savings were shared between providers and insurers, illustrating an early shared savings model. Figure 3 provides a visual overview of the contributing organizations (squares), their roles, interaction (arrows), and an example of CDSS (circle). Data from multiple national registries are integrated by technology partners (double-headed arrow) and linked to GP electronic health records and the GP (single-headed arrow), where CDSS are embedded to support clinical decisions. Finally, CDSS are evaluated with insurers and academic partners. The figure also includes an example of a cardiovascular risk management rule, showing how data generated by GPs trigger actions for patient follow-up, supported by continuous evaluation and feedback between organizations.

From the outset, CDSS and infrastructure development were co-funded by health care insurers and technology partners, who believed in the potential of CDSS and reimbursed GPs for using CDSS, even in the absence of traditional scientific validation. Gaining internal support within the insurer required ongoing efforts from the individual involved in GzGr from the start, who actively used the shared mission to convince colleagues of the value of GzGr:

*We have invited a colleague working at the healthcare insurer, because internal support [for GzGr at the insurer] was needed to keep justifying, once again, why funding should continue.*

[Strategic adviser, regional network, and consulting organization]

To increase the impact of the CDSS, GzGr sought to expand the collaboration with additional GP care groups in the region. However, not all care groups were willing to experiment and change their internal processes to accommodate the CDSS. Therefore, GzGr decided to prioritize GP practices with an “innovative mindset” and willingness to experiment. Additionally, not all collaborations within GzGr endured. A regional hospital withdrew after the departure of the executive initiating GzGr, which respondents linked to a misalignment between individual commitment and organizational priorities.

To conclude, GzGr operationalized their broad mission to enhance preventative care by developing and implementing CDSS. The first mechanism was used: enacting an interorganizational value model, which built on shared value and accountability. We identified two sequential patterns of actions, forming this mechanism: (1) forming a coalition of the willing that shared a common mission, combined their expertise, and mobilized resources; and (2) defining principles for organization selection to ensure mutual value, emphasizing innovation, compliance, cost-plus funding model, and mission alignment. Table 1 presents the sequential patterns of actions that form the mechanism.

### Period 2 (2015-2017): Develop Understandable Algorithms

In the second period, GzGr further developed the interorganizational value model by aligning on development principles, preventing disagreements during or after the CDSS development. Management, together with end users, developed these principles to ensure broad implementation, feasibility, and usability in multiple levels within the organizations. The principles for developing CDSS were as follows.

First, agreement was needed on the pricing structure and ownership of CDSS. For GPs and the insurer, it was not acceptable to invest their time and share sensitive data, while the technology partners would make high profits, own CDSS, and if prices would fluctuate. At the same time, the technology partners needed to work for profit to survive. Eventually, it was agreed that the technology partners were allowed to make a small profit based on a cost-plus price model and that CDSS would be free of property rights:

*They [technology partners] need to make profit otherwise the company will not survive. But we discussed we do not want value-based pricing [assessing the price based on the costs the technology saves], but a cost-plus model...so we can set a fixed price per patient and make it more attractive for the healthcare insurers because the prices are predictable.*

[Innovation manager, technology partner 3]

Second, GzGr fundamentally chose co-creation as the basis for CDSS development, ensuring that CDSS were driven by clinical problems that were prioritized by both GPs, practice nurses, and patients. While clinicians identified problems in practice related to delivering care, technology developers explored what was technically feasible, and funders assessed financial viability. This ensured the integration of the requirements from people, technology, and business. Initially, this human-centered, problem-driven approach challenged technology partners, who were used to a supplier-client model with predefined requests. Now, they had to share responsibility with end users in identifying clinical problems, requiring them to adapt their development processes:

*We apply a problem-driven principle, and because we develop together, you can feel a real drive to actually use it [CDSS] and make the most of it.*

[Director primary care, technology partner 4]

Third, the GzGr organizations agreed that transparency of CDSS and its development was key*.* CDSS’ decision paths (even for AI, using logistic regression) could be followed by GPs, and the functionality was made explainable, allowing GPs to verify accuracy and build confidence in the use of CDSS. This transparency supports GPs in meeting legal requirements that state that health care professionals must be able to explain clinical decisions.

Fourth, aligning with legal, regulatory, and clinical guidelines was essential for GzGr. This meant that GPs retained control over clinical decision-making, with the ability to override CDSS recommendations. Although aligning with guidelines was costly, it ensured quality, scalability, and support from GPs:

*I am pleased with...legal and regulatory guidelines, what might sound paradoxical, but it forces you to demonstrate how your algorithm works,...leading to transparency.*

[Innovation manager, technology partner 3]

Fifth, it was agreed that seamless workflow integration was essential by embedding CDSS in GP electronic health records to avoid system switching. The coalition of the willing convinced the largest GP information system developer (technology partner 4) to join, which made it possible that CDSSs could be integrated within their systems. Respondents noted that achieving technical integration was a significant but successfully managed challenge, as they had experienced how a nonintegrated system was rarely used:

*The initial PDFs were barely used, people had to open a PDF, log into a new system again. This just did not work in practice.*

[Account manager, health care insurer]

While following these 5 principles, 7 CDSS were developed in the GP landscape and were used by 160 GP practices, reaching up to 550,000 patients. An additional 5 CDSS were under development and were tested in 1 to 5 practices, with a reach of 10,000 to 25,000 patients (in 2022; Textbox 1). The development of CDSS started in 2014 and is ongoing.

Textbox 1. **Overview of the 7 developed and 5 clinical decision support systems (CDSS) under development in 2022, based on the aligned development principles, including adoption metrics (number of general practitioner [GP] practices and patients).**

Developed by *Gezonde zorg, Gezonde regio*

- CDSS applications

- Patients at risk of being unserved in cardiovascular risk management.
- Referral protocol for patients with chest pain.
- Feedback on chest pain referral practices.
- Referral protocol for peripheral arterial disease (PAD).
- Peripheral arterial disease: case identification and follow-up.
- Feedback on referral behavior in PAD cases.
- Assessment of indication for goals-of-care conversations in frail older adults.

- Adoption metrics

- Developed CDSS were implemented in 160 GP practices, reaching approximately 550,000 patients.

Development by GzGr

- CDSS applications

- Patients with chronic obstructive pulmonary disease potentially overtreated with inhaled corticosteroids.
- Patients on direct oral anticoagulants/non–vitamin K antagonist oral anticoagulants with chronic kidney disease (chronic kidney disease epidemiology collaboration) for over 12 months.
- Ensuring appropriate laboratory monitoring frequency for patients on central nervous system medications.
- Treatment adjustments based on a significant decline in kidney function.
- Identification of lithium users at high risk of toxicity.

- Adoption metrics

- CDSS under development were implemented in 1 to 5 practices, reaching between 10,000 and 25,000 patients.

In conclusion, the organizations concretized the interorganizational value model by aligning early on developing principles (the sequential process): (1) agreements on ownership and pricing, (2) co-creation, (3) transparency and guideline alignment, and (4) embedding CDSS in daily workflows. These principles translated the coalition’s mission into daily practice, helping organizations navigate conflicts, build trust, and coordinate decisions while preserving their individual and regional value. Additionally, the mechanism of iterative, co-creative experimentation emerged with the first prototypes, driven by structured feedback loops between end users, developers, and decision-makers (the sequential process). The sequential patterns of actions that form these mechanisms are presented in Table 1.

### Period 3 (2018-2021): Implement CDSS in Daily Practice

After the first CDSS was developed, it became clear that the use of CDSS by GPs was lower than initially expected. In response, the focus shifted to integrating CDSS into daily practice and optimizing use, learning from previous experience, and iteratively adapting the strategy, with the same mission.

Initially, during implementation, primary care groups managed communication within their own organizations, but this proved challenging and ineffective. As a result, GzGr started developing materials, webinars, and introduced on-site implementation staff to strengthen end user skills and integration into workflows. Identifying relevant problems for CDSS also remained difficult, leading to the formation of an editorial committee, comprising technology partners, the insurer, and innovative GPs and assistants. They identified widely experienced implementation problems, composed solutions and an implementation plan, and promoted CDSS use among peers.

As the health care insurer and technology partners had substantially invested in the development and implementation, they wanted a guarantee that GPs used CDSS. Therefore, implementation outcomes (eg, CDSS usage rate and adoption of regional GP practices) were developed and agreed upon, obliging the GzGr organizations to act when algorithm usage rates dropped below a certain threshold:

*It’s no longer optional to use the algorithms [CDSS]. We have invested significantly, and the strategic goals must be met. While some front runners are making real progress, others still hesitate. That is why we are now fully focusing on implementation,...healthcare organizations must take responsibility for internal adoption.*

[Account manager, health care insurer]

Additionally, in this period, practice-oriented academic partners joined GzGr to facilitate research, testing, and evaluation, without losing momentum and to ensure a pragmatic process. The results demonstrated that CDSS contributed to time efficiency, improved care quality, and increased adoption by bringing a scientific perspective. The steering committee assessed implementation outcomes across people, technology, and business to ensure stakeholder value. Values were not only monetary; namely, for a GP, saving time was essential, and the health care insurer had to fulfill their duty of care.

GzGr used the implementation and scientific outcomes and user feedback for a continuous iterative learning and improvement experimentation process to refine both the CDSS and the collaboration. The process was as follows: every first version of CDSS is tested in 1 or 2 GP practices (serving between 2000 and 5000 patients). The first tests were done in 5 to 10 GP practices (serving between 10,000 and 25,000 patients). When a CDSS was approved by GzGr, it was implemented in all practices (serving more than 500,000 patients). This process led to adaptations in both the technology and the mission-driven collaboration. Early feedback enabled timely adjustments, strengthened collaboration, and increased support among end users. Respondents noted that this iterative process became a functioning “engine for innovation” within and between the development and implementation phase: creating the opportunity and confidence to develop other technologies and expand the collaboration with a hospital and more care groups.

To conclude, the mechanism “iterative co-creative experimentation” further developed as GzGr shifted from developing technology to embedding CDSS in daily practice. The sequential patterns of actions were (1) co-creation in structured feedback loops, ensuring that people, technology, and organizational perspectives directly informed further development; (2) GzGr took ownership of communication; and (3) technological adjustments were tied to measurable, scientific outcomes. Together, these sequential patterns of actions strengthened shared commitment to GzGr’s mission, while the interorganizational value model streamlined decision-making and maintained focus during iterative development and implementation cycles. Table 1 presents the sequential patterns of actions that shape the mechanisms.

## Discussion

### Principal Study Findings

We conducted a qualitative process analysis of our exploratory case study data to identify which mechanisms helped overcome challenges in the development and implementation of CDSS within GzGr, an interorganizational collaboration. This analysis shed light on sequential patterns of actions by identifying the mechanisms through which challenges were addressed across 3 subsequent periods. We identified 2 mechanisms: (1) enacting an interorganizational value model and (2) iterative, co-creative experimentation. The 3 periods show that the mechanisms reinforced each other and how the underlying actions built on each other over time. Iterative, co-creative experimentation refined the interorganizational value model. In turn, this shared value model made iterative experimentation more focused and effective.

### Comparison With Prior Work

We elaborate on how these mechanisms that explain CDSS can be developed and implemented in an interorganizational context and compare them to previous research.

#### Enacting an Interorganizational Value Model for Mission-Driven Interorganizational Collaboration and CDSS Development

One of the key perceived challenges by GzGr in developing and implementing CDSS across multiple organizations was the fragmentation of responsibilities, interests, and values. This reflects the complexity of the setting, characterized by many independent yet interdependent organizations. GzGr addressed the setting’s complexity by forming an interorganizational value model. Underpinning this value model was a mission-driven coalition of the willing. This coalition enabled GzGr to move beyond organizational silos and foster collaboration by spreading the shared mission and value across individual organizations. Earlier research shows that collaborative performance tends to be strongest when a closely connected group with clear leadership and a shared purpose works together, supported by wider networks that enable collaboration across boundaries [37]. Additionally, GzGr showed that early alignment on shared principles is key to addressing implementation challenges. Agreements about ownership and pricing, transparency, and traceability of CDSS, and embedding them into daily workflow are earlier mentioned in single-organizational literature as facilitating the development and implementation of digital health interventions [16,20]. However, existing literature does not explain how shared principles can be operationalized early into an interorganizational value model, which GzGr’s sequential patterns of actions address.

Embedding people, technology, and organizational interests within this value model enabled GzGr to balance diverse values, address challenges in the primary care process, ensure technical feasibility, and maintain financial viability. Specifically, different organizations and different levels within organizations operate with their own structures, priorities, and financial logic, leading to differences in the perceived value of the collaboration and outcomes [38]. Taking all organizational values into account will help identify priorities, problems, and solutions that can improve real-world implementation of contextually adapted technologies [13,18].

In addition, GzGr continuously assessed value, monetary, or otherwise (such as improved knowledge, diagnostics, or patient care) to enact the interorganizational value model. An earlier study showed that moving beyond monetary value and keeping people, technology, and business in mind can lead to alternative, sustainable payment models [39]. Literature also highlights the importance of integrating people and technology, such as aligning workflows to improve eHealth usability [1,40], but often overlooks the business dimension. While business model design based on desirability, feasibility, and viability of interventions is seen as key to success [13,18,41], financial interdependencies between organizations are seen as a problem for business modeling [12].

The contribution of our study lies in identifying the sequential patterns of actions for enacting an interorganizational value model. This model integrates people, technology, and business perspectives to align principles for CDSS development and guide coordinated actions across independent organizations.

#### Iterative, Co-Creative Experimentation That Spans Development and Implementation

CDSS are considered complex interventions due to the multiple interacting components and the connection between end users and their context. Developing and implementing a complex intervention in a complex system requires an iterative process of development and implementation [10]. In this research, we found that the boundary between development and implementation was blurred and that iterative experimentation was an ongoing process of the sequential patterns of actions. This led to GzGr building and sustaining the involvement of multiple organizations, instead of only one-off interactions between developers and users.

For GzGr, the learning process took place in small-scale, short-cycle experiments with multilevel stakeholders, which is not common in the implementation in Dutch health care. Small-scale, short-cycle testing is especially relevant for eHealth technologies, which evolve rapidly and require adaptation to social and contextual factors for adoption [42]. Therefore, scholars promote small-scale iteration and agile methods such as “fail early, fail often,” without long-term, extensive datasets, to reduce delays and costs [12,41,43]. However, such an iterative approach conflicts with traditional development and lengthy outcome measure processes in the health care industry (eg, medical device regulations or randomized controlled trials). Health care is considered a risk-averse and regulated industry [43]. GzGr involved practice-oriented academic partners, which can foster implementation in a risk-averse industry [44].

Another difference between the technological and health care industries is how implementation is perceived. In the digital industry, implementation is often seen as the final phase and end point, whereas in health care, it is a complex, ongoing process and should not be treated as the last step [36]. We advocate for the mechanism of iterative, co-creative experimentation that bridges development and implementation, rather than traditional, phased models. The National Health Service also implemented a continuous learning and adapting iterative process, aligning the technology with adopters, which requires iterative refinement and an agile approach, leading to regional adoption [45].

In such an iterative process (rather common in digital innovation in other industries [46]), it is crucial to involve all relevant stakeholders in the design and test phase to improve the implementation success of digital health interventions, realizing a co-creative process [1,40,47,48]. By involving end users in the development, a collaboration can anticipate implementation problems. Additionally, consensus on CDSS functionality and added value supports implementation [48,49]. The sequential pattern of actions included user feedback, which led to rapid technological improvements, strengthening trust in both the mission and the collaboration before broader implementation to more risk-averse groups. This method is in line with AI adoption within the National Health Service [20].

Previously mentioned studies mainly emphasized end user involvement (particularly health care professionals) during development in controlled settings. However, involving multilevel stakeholders during this process helps generate buy-in by aligning intervention design with strategic goals, thereby supporting adoption and keeping service models central [1,13,41,47,50,51]. In doing so, GzGr illustrates how to overcome the common pitfall of “pilotitis,” where digital health innovations stall in the pilot phase without scaling [52].

Our findings contribute by identifying the sequential patterns of actions that underlie an iterative, co-creative experimentation in a risk-averse setting. GzGr created a continuous, co-creative experimentation process spanning development and implementation and sustainably involving multilevel stakeholders, who kept the mission in mind and did not treat development and implementation as separate phases. This illustrates how joint progress can be achieved by connecting social change and technological development, which was crucial for achieving sustainable implementation of CDSS.

### Limitations

To interpret the results of this study, we have reflected on several limitations in our research. First, the focus on a single case in the Netherlands restricts the generalizability of the findings to other contexts. CDSSs have been scaled to other organizations and are currently being expanded to additional regions. However, it remains uncertain to what extent the collaboration’s approach to CDSS development and implementation is dependent on context or how this compares to other countries. Further research is needed to validate these findings across different organizations, regions, and countries.

Second, reflecting on our use of qualitative data and a retrospective analysis, recalling bias and interpretative variation among respondents might exist. We mitigated this limitation by combining data sources (interviews, documents, and GzGr experts), making use of data triangulation. Moreover, our transdisciplinary research team enriched qualitative data analysis by incorporating diverse perspectives, including medical expertise, innovation ecosystems, organizational governance, design, and implementation. This multiperspective approach enhances the study’s applicability and relevance, despite its single-case focus. Additionally, the retrospective character of this research gave us the opportunity to compose a chronological overview. Due to this nature, the study has a descriptive rather than a predictive value, as formally no causality between actions, sequences of actions, and effects was assessed. Future research may provide predictive insights into how the timeline of the sequential patterns of actions and mechanisms unfolds during implementation.

Third, our identification of 2 mechanisms may reflect a degree of interpretation of the researchers, as is inherent to qualitative research. We addressed this by documenting our observations in a chronological overview table (Multimedia Appendix 3), performing member checks, and linking our findings to existing literature. These factors provide a thorough understanding of the mechanisms underlying CDSS development and implementation in an interorganizational context.

### Implications and Future Research

This research identified mechanisms that help interorganizational collaborations overcome challenges in the iterative development and implementation of CDSS. In this perspective, our study extends the determinant-level frameworks NASSS, Centre for eHealth and Wellbeing Research, and UK Medical Research Council by adding an interorganizational, process-level perspective that captures how collaboration unfolds across independent organizations. It shows how organizations, while maintaining their own interests, continuously realign around a shared goal through iterative steps to compose an interorganizational value model that sustains collective progress.

Our practical implications consist of 3 elements. First, to navigate complexity, the development and implementation of CDSS in interorganizational collaborations should involve multilevel stakeholders from the start of the collaboration, especially innovative and influential individuals who are willing to go beyond formal roles to advance shared goals and form a coalition of the willing. Second, an orchestrating actor, such as GzGr, is needed to facilitate communication, alignment, and technical support across individual organizations within interorganizational collaborations. Third, an iterative approach should be adopted, where feedback from multilevel stakeholders informs continuous technological and collaborative improvements that will support both adoption and long-term collaboration.

Future research should enhance the generalizability of our findings by comparing this case with other interorganizational collaborations to identify shared mechanisms. Additionally, further investigation is needed into the scalability of CDSS, distinguishing context-dependent elements. Finally, future research should explore how these mechanisms can be systematically applied to support adaptive implementation in research and practice.

### Conclusions

Our study offered insights into the mechanisms for developing and implementing CDSS in an interorganizational collaboration in primary care. Our findings extend the determinant-level implementation frameworks for complex digital health interventions by offering process-level insights into sequential patterns of actions that show how change unfolds in complex, real-world settings. We identified two mechanisms: (1) enacting an interorganizational value model and (2) iterative, co-creative experimentation. These mechanisms supported both technological and organizational change, through the approach of integrating people, technology, and business aspects for all and across organizations. Through this approach, CDSS were generated and implemented via an iterative, co-creative experiment. This resulted in CDSS that were (financially) viable and that generated added value per individual stakeholder and organization. Additionally, CDSS could be embedded in the individual organizations as they fulfilled their values, needs, and interests. Therefore, CDSSs reached multilevel support and were well adopted.

Overall, our study enhances the understanding of how sequential patterns of actions enable digital health innovations to be embedded and sustained in an interorganizational collaboration. It highlights that successful implementation in complex systems depends on establishing shared principles early, forming the basis for an interorganizational value model. Simultaneously, an iterative collaboration spanning development and implementation is required. Throughout this process, continuous alignment of social, technological, and organizational dimensions is essential. We offered both practical and conceptual contributions to understanding how digital health interventions can be embedded and sustained in interorganizational collaboration.

## Supplementary material

10.2196/83084 Multimedia Appendix 1 Overview of the participants.

10.2196/83084 Multimedia Appendix 2 Elaboration on coding and codebook process.

10.2196/83084 Multimedia Appendix 3 Chronological overview of events, challenges, and mechanisms encountered by *Gezonde zorg, Gezonde regio*.

10.2196/83084 Checklist 1 COREQ checklist.

## ABBREVIATIONS

CDSS: clinical decision support systems
COREQ: Consolidated Criteria for Reporting Qualitative Research
GP: general practitioner
GzGr: *Gezonde zorg, Gezonde regio* (Healthy care, Healthy region)
NASSS: Nonadoption, abandonment, scale-up, spread, and sustainability
