# Supplementary material for: Interorganizational Mechanisms for Developing and Implementing Clinical Decision Support Systems in Primary Care: Exploratory, Qualitative Case Study
Source: J Med Internet Res. 2026 Mar 5;28:e83084. doi: 10.2196/83084 (PMC12978902; doi:10.2196/83084)
Supplement: Multimedia Appendix 1 [file jmir-v28-e83084-s001.docx]

Appendix II: an overview of the participants

| **Identification number** | **Organization** | **Job title** |
| --- | --- | --- |
| 1 | Regional network and consulting organization | Strategic adviser |
| 2 | Technology partner 1 | Innovation manager |
| 3 | National health insurance umbrella organization | Policy advisor |
| 4 | Technology partner 3 | Data scientist |
| 5 | Technology partner 4 | Innovation manager |
| 6 | Technology partner 4 | Director |
| 7 | Technology partner 2 | Professor of data science |
| 9 | University medical center | Physician |
| 10 | Healthcare insurer | Account manager |
| 11 | Technology partner 4 | Director primary care |
| 12 | Care group 2 | General practitioner and director |
| 13 | Technology partner 1 | Executive director |
| 14 | University medical center | Cardiologist |
| 15 | Patient representation | Executive |
