# Supplementary material for: Interorganizational Mechanisms for Developing and Implementing Clinical Decision Support Systems in Primary Care: Exploratory, Qualitative Case Study
Source: J Med Internet Res. 2026 Mar 5;28:e83084. doi: 10.2196/83084 (PMC12978902; doi:10.2196/83084)
Supplement: Multimedia Appendix 2 [file jmir-v28-e83084-s002.docx]

Appendix IV: elaboration on coding process and codebook

Coding process:

(i) JS and JvW performed a round of open coding using recognition words that indicate time (such as last year, at the start, earlier) to identify and code events. We mapped these events over time and compared the findings with the retrieved documents, to triangulate the ordering of events. We then defined periods, referring to a specific segment in time of implementation and/or development

(ii) We performed a second round of open coding of the transcripts, this time focusing on identifying how GzGr reacted to emergent developments (such as dealing with unexpected outcomes, low algorithm usage, delays, and issues involving specific participating organizations).

| **Theme** | **Code** | **Definition** |
| --- | --- | --- |
| Compliance with laws and regulations | +Privacy | Technology and collaboration comply with the GDPR; personal data are well protected and treated confidentially, which facilitates implementation. |
| Compliance with laws and regulations | -Privacy | Technology and collaboration do not comply with the GDPR; personal data is less well protected and treated less confidentially, which hinders implementation. |
| Compliance with laws and regulations | +Security | Compliance with Dutch legal security requirements (e.g., proper certification such as ISO/NEN, logging, quality standards), facilitates the implementation of collaboration and technology. |
| Compliance with laws and regulations | -Security | Fail to comply with Dutch legal security requirements (e.g., proper certification such as ISO/NEN, logging, quality standards) hinders the implementation of collaboration and technology. |
| Compliance with laws and regulations | +Demonstrating effectiveness | Demonstrating the effectiveness of technology and collaboration facilitates implementation. Effectiveness means that technology or collaboration delivers the same or improved health outcomes at lower costs and with less time investment. |
| Compliance with laws and regulations | -Demonstrating effectiveness | Not demonstrating the effectiveness of the technology and collaboration hinders implementation. |
| Market mechanisms | +Role of government | The government takes an active role in the collaboration or development of technology. This role facilitates the implementation of both technology and collaboration. |
| Market mechanisms | -Role of government | The government takes an inactive role in the collaboration or development of technology. This role hinders the implementation of both technology and collaboration. |
| Market mechanisms | +Competition | Competition facilitates the implementation of collaboration and technology. |
| Market mechanisms | -Competition | Competition hinders the implementation of collaboration and technology. |
| Financing is essential | +Regular funding | Regular funding streams facilitate the implementation of collaboration and technology. |
| Financing is essential | -Regular funding | Regular funding streams hinder the implementation of collaboration and technology. |
| Technology development | +Development | The development of technology facilitates implementation. Technology also meets preconditions that support implementation. |
| Technology development | -Development | The development of technology hinders implementation. Technology does not meet preconditions, which hampers implementation. |
| Technology development | +Design | The applied design principles facilitate the implementation of the technology. |
| Technology development | -Design | The applied design principles hinder the implementation of the technology. |
| Implementation | +Accessibility | The technology is easy to use and simple to understand for end users, which facilitates implementation. |
| Implementation | -Accessibility | Technology is not easy to use and less simple to understand for end users, which hinders implementation. |
| Implementation | +Trust end users (healthcare professionals, patients) | End users’ trust in technology and collaboration facilitates implementation. Trust may result from satisfaction and confidence in the technology. |
| Implementation | - Trust end users (healthcare professionals, patients) | Lack of end users’ trust in technology and collaboration hinders implementation. |
| Implementation | +Training | Strengthening end users’ skills in using the technology and collaboration facilitates implementation. |
| Implementation | -Training | Not strengthening end users’ skills in using the technology and collaboration hinders implementation. |
| Implementation | +Willingness to change | End users are willing to change, which facilitates the implementation of technology and collaboration. They are positively inclined toward technology and collaboration and have confidence in successful implementation. |
| Implementation | -Willingness to change | End users are not willing to change, which hinders the implementation of technology and collaboration. They are not positively inclined toward technology and collaboration and have no confidence in successful implementation. |
| Implementation | +Evaluation | Systematically monitoring the use of the technology and continuing development facilitates implementation. |
| Implementation | -Evaluation | Not systematically monitoring the use of technology and not continuing development hinders implementation. |
| Collaboration | +Infrastructure | A data infrastructure between organizations facilitates the implementation of the technology. |
| Collaboration | -Infrastructure | A data infrastructure between organizations hinders the implementation of the technology. |
| Collaboration | +Trust within GzGr | Participants trust each other (the individuals involved), which facilitates the implementation of the collaboration. |
| Collaboration | -Trust within GzGr | Participants do not trust each other (the individuals involved), which hinders the implementation of the collaboration. |
| Collaboration | +Mission/vision | GzGr has a clear mission/vision (future goals and how to achieve them) that is communicated and embraced by participants, which facilitates the implementation of the collaboration. |
| Collaboration | -Mission/vision | GzGr does not have a clear mission/vision (future goals and how to achieve them) that is communicated and embraced by participants, which hinders the implementation of the collaboration. |
| Collaboration | +Innovators | The collaboration includes participants who generate and implement innovative ideas, which facilitates the implementation of the collaboration. |
| Collaboration | -Innovators | The collaboration does not include participants who generate and implement innovative ideas, which hinders the implementation of the collaboration. |
| Collaboration | +Participating parties | Participants from the participating organizations are actively engaged, communicate well with each other, and contribute constructively, which facilitates the implementation of the collaboration. |
| Collaboration | -Participating parties | Participants from the participating organizations are not actively engaged, do not communicate well with each other, and do not contribute constructively, which hinders the implementation of the collaboration. |
| Collaboration | +Communication | Clear and transparent communication about technology and collaboration to end users facilitates implementation. |
| Collaboration | -Communication | Lack of clear and transparent communication about technology and collaboration to end users hinders implementation. |
| Collaboration | + Investment by participating organizations | The participating parties invest time and money, enabling collaboration and technology to be implemented, which facilitates implementation. |
| Collaboration | - Investment by participating organizations | The participating parties invest less time and money, which hinders the implementation of collaboration and technology. |
| Scaling up | +Scaling up GzGr | Opportunities for scaling up facilitate the implementation of the collaboration. |
| Scaling up | -Scaling up GzGr | Limited opportunities for scaling up hinder the implementation of the collaboration. |
| Scaling up | +Scaling up technology | Opportunities for scaling up facilitate the implementation of the technology. |
| Scaling up | -Scaling up technology | Limited opportunities for scaling up hinder the implementation of the technology. |
| Scaling up | +Cost savings | Technology and collaboration results in reduced health care costs, which facilitates implementation. |
| Scaling up | -Cost savings | Technology and collaboration do not result in reduced health care costs, which hinders implementation. |
| Scaling up | + Proving added value | The added value of the technology and collaboration (such as time savings, increased data/knowledge, reduced workload) is clear to the end user, which facilitates implementation. |
| Scaling up | - Proving added value | The added value of the technology and collaboration (such as time savings, increased data/knowledge, reduced workload) is not clear to the end user, which hinders implementation. |
