# Supplementary material for: Interorganizational Mechanisms for Developing and Implementing Clinical Decision Support Systems in Primary Care: Exploratory, Qualitative Case Study
Source: J Med Internet Res. 2026 Mar 5;28:e83084. doi: 10.2196/83084 (PMC12978902; doi:10.2196/83084)
Supplement: Multimedia Appendix 3 [file jmir-v28-e83084-s003.docx]

Appendix III: a chronological overview of events, challenges, and mechanisms

NB. The two mechanisms were: (1) Enacting an interorganizational value model, and (2) iterative, co-creative experimentation.

| **Period** | **Challenge** | **Actions** | **Relations to mechanism** |
| --- | --- | --- | --- |
| 2010 – 2014: setting up collaboration | Involving organizations and innovative people who share the same ideals and principles | Started the collaboration with influential individuals of the largest regional healthcare insurer executives from a primary care group and a regional hospital wanting to realize population funding | Started change mechanism 1 |
|  | Discover what is technologically necessary to realize mission | Research institute participated in collaboration for measuring baseline population funding | Leverage mechanism 1 |
|  | Outcome of research institute was not as expected; collaboration stopped with research institute |  |  |
|  |  | Mission changed to enhancing preventative care interventions in GP practices by developing and implementing CDSS | Leverage mechanism 1, setting up principles |
|  | Involving organizations and innovative people who share the same ideals and principles | New technology partner joined the collaboration as trusted third party for standardizing and integrating data used in research | Leverage mechanism 1 |
|  | Involving organizations and innovative people who share the same ideals and principles | Other technology partners joined the organization based on principles | Leverage mechanism 1, setting up principles |
|  |  | Setting up data infrastructure was partly subsidized by innovation funds of healthcare insurers | Leverage mechanism 1, setting up principles |
|  |  | Developing data infrastructure between participating organizations | Leverage mechanism 1, development according to principles |
|  |  | Individual organizations partially financed resources themselves, and health insurers financed GPs for using CDSS, which was partially used for development CDSS | Result of mission (mechanism 1) |
|  |  | CDSS research and development started based on data analysis | Leverage mechanism 1, development according to principles |
|  |  | First positive outcomes of CDSS, e.g., changing treatment for patients with elevated cholesterol, leading to more health gains and safer treatment for patients and cost saving, and more support for CDSSs | Enhanced mechanism 1 |
|  | Not entire care groups are innovative, but individual GP practices are. | Shift in focus to involving innovative GP practices (to convince others of the positive effects) | Leverage mechanism 1 |
|  | Director of healthcare organization, advocate of collaboration, left organization and the hospital quit the collaboration, because of privacy and transparency reasons |  |  |
| 2015 – 2017: develop and implement understandable CDSS | Collaborating with new partners that align with the expectations and needs of GzGr | Setting up development principles to ensure progress to GzGr their mission | Leverage mechanism 1 |
|  | Developing understandable CDSS | Focus on building understandable CDSS: developing CDSS from primary process, using feedback from end-users (structuring data and developing algorithm) | Leverage mechanism 1, development according to principles |
|  |  | Developing CDSS from primary process, using feedback from end-users (structuring data and developing algorithm) | Leverage mechanism 1, development according to principles and iterative testing and evaluation, starting mechanism 2 |
|  | Technology partner has a demand-driven model, resulting in also developing requests which are not widely experienced as problem | Technology developers changed processes to developing from primary process | Leverage mechanism 1 |
|  | No seamless workflow integration, signals of CDSS communicated through pdf. | New technology partner (GP information system developer) joined collaboration, ensuring an integration of CDSS in GP information system | Leverage mechanism 1 |
|  |  | Started process for MDR certification | Leverage mechanism 2 |
|  |  | Medical ethical protocol compiled by academic hospital | Leverage mechanism 2 |
|  |  | 6 knowledge rules deployed, 5 in development | Result of mechanism 1 and 2 |
| 2018 – 2021: focus on implementing CDSS in daily practice | Use of CDSS low | More focus on implementation by targeted support and creating urgency | Leverage mechanism 2 |
|  | Improve CDSS, develop new infrastructure and integrating new data of different partners (GP information system, pharmacy) | Further develop existing CDSS, not designing new ones | Leverage mechanism 2 |
|  | Internal communication is responsibility of own organizations | GzGr improved information material, developed training, and informed community actively. | Leverage mechanism 2 |
|  |  | New partners (care groups) joined the collaboration, expanding the service area | Result mechanism 1 |
|  | Shift of focus from development to implementation | Established editorial committee (consist of GPs, GP assistants, technical designers, and healthcare insurers) | Leverage mechanism 2 |
|  | Shift of focus from development to implementation | Specific implementation staff assigned, to improve digital skills of healthcare professionals | Leverage mechanism 2 |
|  | Improve CDSS | Testing algorithm prototypes and retrieving feedback from GPs, the positive feedback increased support for collaborative mission | Leverage mechanism 2 and enhance mechanism 1 |
|  |  | More commitment of technology partners for collaboration | Outcome mechanism 2 and enhance mechanism 1 |
|  |  | Frequency of data delivery changed, so GPs are not overwhelmed | Leverage mechanism 2 |
|  |  | Developed dashboard with insights into the use of CDS CDSS | Leverage mechanism 2 |
|  |  | Strategic dialogues with directors and IT specialists about the learning process and the opportunities of GzGr for individual organizations | Leverage mechanism 2 and enhance mechanism 1 |
